# Perceived barriers and facilitators to health behaviors in European childhood cancer survivors: A qualitative PanCareFollowUp study

**DOI:** 10.1002/cam4.5911

**Published:** 2023-04-07

**Authors:** Eline Bouwman, Saskia M. F. Pluijm, Iridi Stollman, Vera Araujo‐Soares, Nicole M. A. Blijlevens, Cecilia Follin, Jeanette F. Winther, Lars Hjorth, Tomas Kepak, Katerina Kepakova, Leontien C. M. Kremer, Monica Muraca, Helena J. H. van der Pal, Carina Schneider, Anne Uyttebroeck, Gertrui Vercruysse, Rod Skinner, Morven C. Brown, Rosella P. M. G. Hermens, Jacqueline J. Loonen, the PanCareFollowUp Consortium

**Affiliations:** ^1^ Center of Expertise for Cancer Survivorship, Department of Hematology Radboud University Medical Center Geert Grooteplein Zuid 10 6525 GA Nijmegen the Netherlands; ^2^ Princess Máxima Center for Pediatric Oncology Heidelberglaan 25 3584 CS Utrecht the Netherlands; ^3^ Center for Preventive Medicine and Digital Health (CPD), Medical Faculty Mannheim Heidelberg University Röntgenstraße 7 D‐68167 Mannheim Germany; ^4^ Oncology, Department of Clinical Sciences Lund, Lund University Skåne University Hospital Lasarettsgatan 40 221 85 Lund Sweden; ^5^ Childhood Cancer Research Group Danish Cancer Society Research Center Strandboulevarden 49 2100 Copenhagen Denmark; ^6^ Department of Clinical Medicine, Faculty of Health Aarhus University and Aarhus University Hospital Palle Juul‐Jensens Boulevard 82 8200 Aarhus Denmark; ^7^ Pediatrics, Department of Clinical Sciences Lund, Lund University Skåne University Hospital Lasarettsgatan 40 221 85 Lund Sweden; ^8^ International Clinical Research Center (FNUSA‐ICRC) at St. Anne's University Hospital Masaryk University Pekařská 53 Brno 656 91 Czech Republic; ^9^ Department of Pediatrics, Emma Children's Hospital Amsterdam UMC Meibergdreef 9 1105 AZ Amsterdam the Netherlands; ^10^ Faculty of Medicine Utrecht University and Utrecht Medical Center Universiteitsweg 98 3584 CG Utrecht the Netherlands; ^11^ DOPO Clinic, Division of Pediatric Hematology and Oncology IRCCS Istituto Giannina Gaslini Via G. Gaslini, 5 16147 Genoa Italy; ^12^ PanCare Jacobus Bellamylaan 16 1401 AZ Bussum the Netherlands; ^13^ Childhood Cancer International ‐ Europe Servitengasse 5/16 1090 Vienna Austria; ^14^ Department of Oncology, Pediatric Oncology KU Leuven Leuven Belgium; ^15^ Department of Pediatric Hematology and Oncology University Hospitals Leuven Herestraat 49 3000 Leuven Belgium; ^16^ Wolfson Childhood Cancer Research Center, Newcastle University Center for Cancer Newcastle University NE1 7RU Newcastle upon Tyne Herschel Building, Brewery Lane UK; ^17^ Great North Children's Hospital Royal Victoria Infirmary Queen Victoria Road Newcastle upon Tyne NE1 4LP UK; ^18^ Wolfson Childhood Cancer Research Center, Translational and Clinical Research Institute Newcastle University Herschel Building, Brewery Lane Newcastle upon Tyne NE1 7RU UK; ^19^ Population Health Sciences Institute Newcastle University Ridley Building 1, Queen Victoria Road Newcastle upon Tyne NE1 7RU UK; ^20^ Scientific Institute for Quality of Healthcare (IQ Healthcare) Radboud University Medical Center Geert Grooteplein 21 6525 EZ Nijmegen the Netherlands

**Keywords:** behavioral science, cancer prevention, cancer risk factors, pediatric cancer, survival

## Abstract

**Background:**

Healthy behaviors, that is, engaging in regular physical activities, maintaining a healthy diet, limiting alcohol consumption, and avoiding tobacco and drug use, decrease the risk of developing late adverse health conditions in childhood cancer survivors. However, childhood cancer survivors may experience barriers to adopting and maintaining healthy behaviors. This study aimed to assess these barriers and facilitators to health behavior adoption and maintenance in childhood cancer survivors.

**Methods:**

A focus group ( *n*  = 12) and semi‐structured telephone interviews ( *n*  = 20) were conducted with a selected sample of European and Dutch childhood cancer survivors, respectively. The Theoretical Domains Framework (TDF) was used to inform the topic guide and analysis. Inductive thematic analysis was applied to identify categories relating to barriers and facilitators of health behavior adoption and maintenance, after which they were deductively mapped onto the TDF.

**Results:**

Ten TDF domains were identified in the data of which “Knowledge,” “Beliefs about consequences,” “Environmental context and resources,” and “Social influences” were most commonly reported. Childhood cancer survivors expressed a need for knowledge on the importance of healthy behaviors, possibly provided by healthcare professionals. They indicated physical and long‐term benefits of healthy behaviors, available professional support, and a supporting and health‐consciously minded work and social environment to be facilitators. Barriers were mostly related to a lack of available time and an unhealthy environment. Lastly, (social) media was perceived as both a barrier and a facilitator to healthy behaviors.

**Conclusion:**

This study has identified education and available professional support in health behaviors and the relevance of healthy behaviors for childhood cancer survivors as key opportunities for stimulating health behavior adoption in childhood cancer survivors. Incorporating health behavior support and interventions for this population should therefore be a high priority.

## Graphical abstract

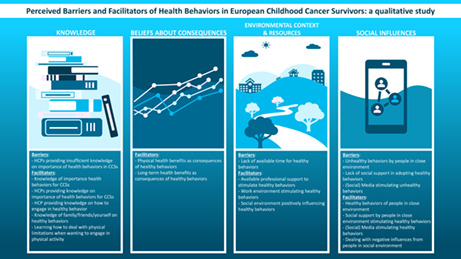

## 1 INTRODUCTION

The last four decades have seen great improvements in childhood cancer survival with 5‐year survival rates increasing from 30% in 1970 to more than 80% at present. Currently, there are over 300,000 and 400,000 childhood cancer survivors living in Europe and the United States, respectively.^1^, ^2^, ^3^, ^4^ However, an unfortunate consequence of cancer treatment is that a majority of survivors are predisposed to an elevated life‐long risk of developing health‐related late effects.^5^, ^6^, ^7^

Previous research has shown that health behaviors may have an impact upon a range of late effects.^8^, ^9^, ^10^, ^11^ For example, several studies have found associations between physical activity and a better cardiometabolic profile in adult survivors of childhood cancer.^8^, ^9^, ^10^, ^12^ In addition, a study by Tonorezos et al. showed that adherence to a Mediterranean diet pattern with high fruit and vegetable intake and low meat intake was associated with better metabolic parameters in adult survivors of acute lymphoblastic leukemia. To be specific, for each point higher on the Mediterranean diet score (range 0–8), the odds of having the metabolic syndrome fell by 31% in this population.^13^ Excessive alcohol consumption is known to further increase the risk of multiple types of cancer, depression, epilepsy, stroke, hypertension, osteoporosis, and liver cirrhosis, whereas smoking may potentiate the organ damage resulting from treatment with radiotherapy or alkylating agents.^14^ Lastly, in the general population, recreational drug use (e.g., cannabis or cocaine) is known to have multiple negative effects on organ systems, including the cardiovascular system.^15^, ^16^ Avoiding unhealthy behaviors as a childhood cancer survivor is therefore favorable to preserve good health.

Given the potential benefits of healthy behaviors, several guidelines provide recommendations for these in childhood cancer survivors, such as engaging in regular physical activity, eating a healthy diet, avoiding tobacco use, limiting alcohol consumption, and refraining from drug use.^17^, ^18^, ^19^ Despite these recommendations, research reveals that survivors often have a poor diet and sedentary behaviors.^20^, ^21^ In addition, 26% of childhood cancer survivors are still tobacco users,^22^ whereas, considering alcohol consumption, 16% and 8% are considered to be risky (≥3 alcoholic drinks per day or ≥7 and 14 per week for women and men, respectively) or heavy (≥5 and 6 alcoholic drinks per day for women and men, respectively) drinkers.^14^ However, survivors may not know knowledgeable about health behaviors and the long‐term health benefits of adherence to these behaviors. Likewise, survivors may be dealing with physical and socio‐economic constraints that may limit or impede participation in physical activity or eating a healthy diet.^21^ Regardless, their cancer history can also make survivors more aware of their health.^23^, ^24^, ^25^ Therefore, survivors may be more motivated to adopt healthy behaviors than their peers.^26^, ^27^

**FIGURE 1:**
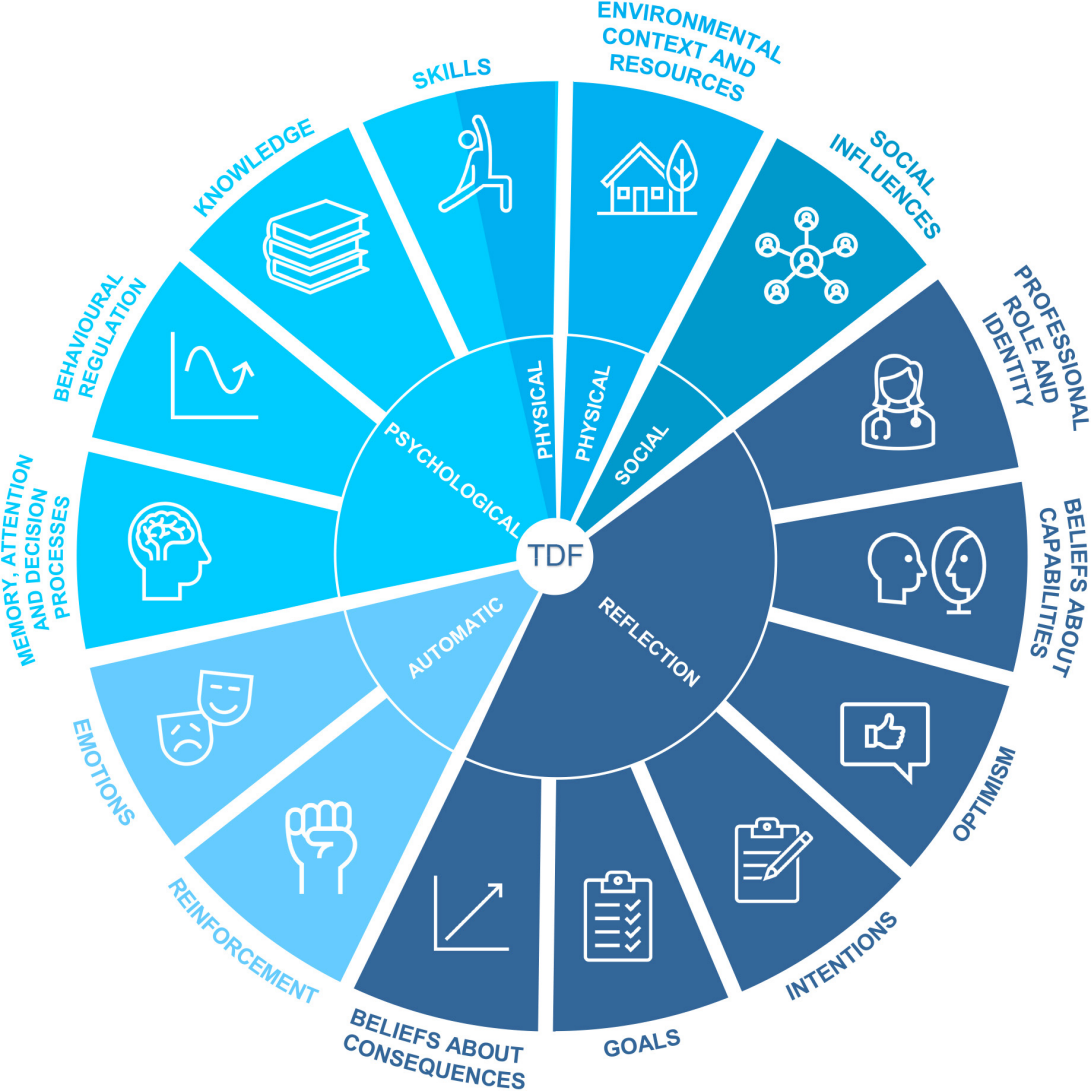
Domains of the Theoretical Domains Framework. Adapted from Cane et al.^28^

Gaining insight into the barriers and facilitators of engaging in healthy behaviors for childhood cancer survivors is essential for intervention development by healthcare professionals involved in survivorship care. In addition, this will lead to the development of appropriate health behavior support provided by healthcare professionals, and will create awareness of health behaviors among childhood cancer survivors. The Theoretical Domains Framework (TDF), a theoretical framework resulting from the synthesis of 128 constructs from 33 behavior change theories, can serve as a means to gain a comprehensive understanding of barriers and facilitators related to the adoption or maintenance of healthy behaviors (Figure 1; Table 1).^28^, ^29^ It has been known that changing behaviors is more effective when interventions are based on evidence‐based principles of behavior change. However, as in the past, it was found to be difficult to identify the specific processes underlying successful behavior change with the existence of so many different behavior change theories and constructs, behavioral scientists and implementation researchers integrated these 128 constructs into 14 domains that may explain behavior and behavior change. These domains were either related to psychological, physical, social, reflective, or automatic influences on behavior implementation and can be used in improving the implementation of interventions or evidence‐based practice. The TDF was applied earlier in a systematic review by Brown et al. to determine barriers and facilitators to physical activity in childhood cancer survivors.^30^

**TABLE 1:** The Theoretical Domains Framework (v2) domains and definitions.^28^, ^29^

Knowledge: an awareness of the existence of something
2 Skills: an ability or proficiency acquired through practice
3 Social/professional role and identity: a coherent set of behaviors and displayed personal qualities of an individual in a social or work setting
4 Beliefs about capabilities: acceptance of the truth, reality, or validity about an ability, talent, or facility that a person can put to constructive use
5 Optimism: the confidence that things will happen for the best or that desired goals will be attained
6 Beliefs about consequences: acceptance of the truth, reality, or validity about outcomes of a behavior in a given situation
7 Reinforcement: increasing the probability of a response by arranging a dependent relationship, or contingency, between the response and a given stimulus
8 Intentions: a conscious decision to perform a behavior or a resolve to act in a certain way
9 Goals: mental representations of outcomes or end states that an individual wants to achieve
10 Memory, attention, and decision processes: the ability to retain information, focus selectively on aspects of the environment, and choose between two or more alternatives
11 Environmental context and resources: any circumstance of a person's situation or environment that discourages or encourages the development of skills and abilities, independence, social competence, and adaptive behavior
12 Social influences: those interpersonal processes that can cause individuals to change their thoughts, feelings, or behaviors
13 Emotion: a complex reaction pattern, involving experiential, behavioral, and physiological elements, by which the individual attempts to deal with a personally significant matter or event
14 Behavioral regulation: anything aimed at managing or changing objectively observed or measured actions

This study aimed to explore childhood cancer survivors' perceived barriers and facilitators to adopting and maintaining healthy behaviors.

## 2 METHODS

### 2.1 Study design

This explorative qualitative study was conducted as part of the European‐wide PanCareFollowUp project and used both telephone interviews and a focus group to explore barriers and facilitators of health behavior adoption. To report this study, the consolidated criteria for reporting qualitative research (COREQ) were used. The procedures were approved by the Research Ethics Committee CMO Arnhem‐Nijmegen (case number 2019‐5630).^31^

### 2.2 Participants

Childhood cancer survivors were eligible if they: (i) were aged ≥18 years; (ii) were in complete remission for cancer; (iii) had been treated with chemotherapy and/or radiation therapy for childhood cancer (i.e., diagnosed ≤18 years of age); (iv) had completed treatment ≥3 years ago; and (v) were in possession of sufficient English or Dutch language skills to take part in the study. To have a broad overview of barriers and facilitators to health behaviors, one focus group with a minimum of five and a maximum of twelve childhood cancer survivors was planned. Recruiting fewer participants would limit the group dynamics and generalizability of the results, whereas recruiting more than 12 participants would limit the participant's opportunity to share their thoughts and opinions. In addition, an initial analysis sample of 20 childhood cancer survivors for the interviews was planned. Given the narrow aim and specificity of this study, the number of participants in the focus group in combination with the 20 planned interviews was thought to be adequate to reach sufficient information power and code saturation.^31^, ^32^ After analysis of the focus group and 20 interviews, saturation would be evaluated by reflecting on the repetition of observed patterns and codes.

### 2.3 Recruitment

Participants were recruited with a purposive sampling method for a focus group with other childhood cancer survivors or an individual telephone interview. The participants for the focus group were recruited after being invited by the European branch of Childhood Cancer International (CCI Europe); a parent and childhood cancer survivor organization. All childhood cancer survivors associated with CCI Europe were provided with information about the focus group in an email sent by a representative of CCI Europe. If interested, they could sign up for this focus group by replying to the representative of CCI Europe. After being provided with the opportunity to ask further questions about the study, written informed consent of the focus group participants was obtained at the start of the focus group. For the individual telephone interviews, childhood cancer survivors were recruited from two Dutch survivorship care centers. Every childhood cancer survivor who met the inclusion criteria was approached and informed about the study by healthcare professionals during his or her regular follow‐up visit at the center. If interested, the childhood cancer survivors received an information package containing an information letter and an informed consent form. When written informed consent was obtained, a researcher (EB or SP) phoned the survivor to provide them with further practical information about the study and to make an appointment for the interviews at a day and time of their convenience.

### 2.4 Procedures

The interviews and focus group were facilitated by a researcher (EB) who is trained in qualitative research with an interest in health behaviors and experience in cancer survivorship research. Two note‐takers (a representative of CCI Europe and a researcher [SP]) was present to take field notes of key points raised.

The interviews and focus group took place between September 2019 and March 2020. All 20 interviews were conducted in Dutch via telephone with childhood cancer survivors. The focus group with European participants took place on‐site during a biannual PanCare meeting in Basel (Switzerland) and was conducted in English. Prior to the interviews, interview participants received an invitation by mail to fill out a questionnaire online via an electronic data capture system (Castor EDC), whereas participants of the focus group were asked to complete the questionnaire on paper at start of the discussion. This questionnaire captured basic demographic information (e.g., country of birth, age, educational level), and medical information (e.g., childhood cancer diagnosis, years since treatment, etc.). At the start of the telephone interviews, participants were asked how they would describe their health behaviors, including physical activity, diet, smoking, and drug and alcohol use. As the focus group concerned a group setting, no questions were asked about describing individual health behaviors to respect the participant's privacy. The interviews and focus group were guided by the same semi‐structured topic guide informed by the TDF (Figure 1; Table 1).^28^, ^29^ The topic guide was pilot‐tested by childhood cancer survivor representatives affiliated with CCI Europe prior to the start of the study. Audio recordings of the interviews and focus group were professionally transcribed verbatim for analysis and anonymized. The average duration of the interviews was 38 min, whereas the focus group lasted 65 min.

### 2.5 Analysis

**FIGURE 2:**
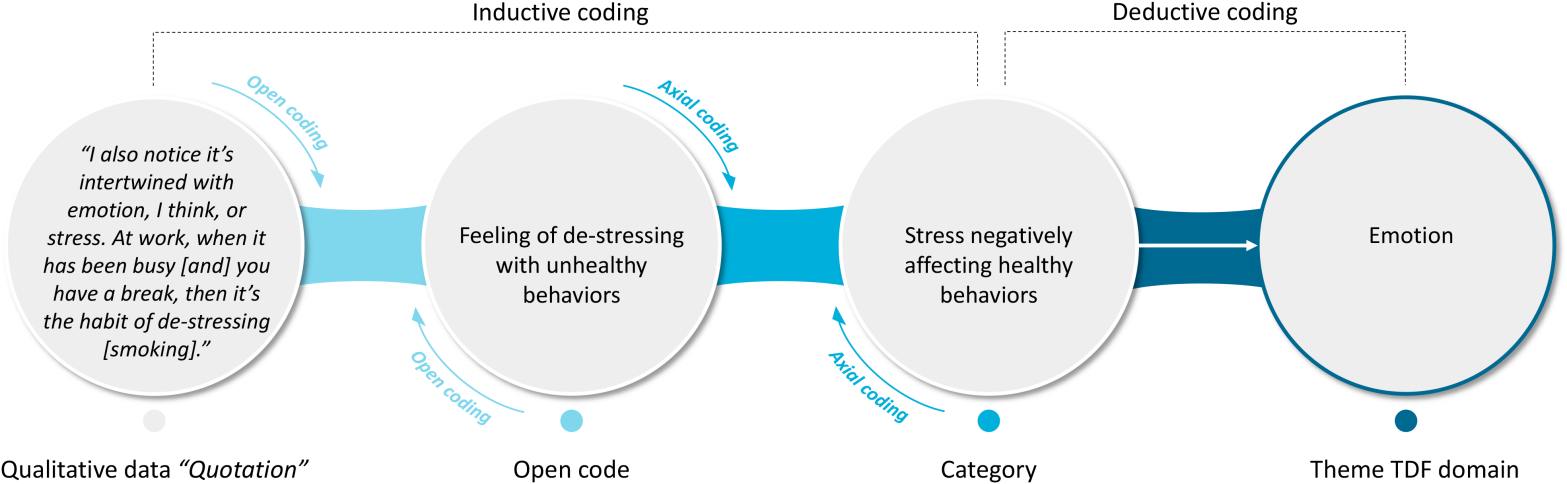
Illustrative example of the coding process.

Analysis of qualitative data was facilitated by the qualitative data analysis software Atlas.ti 8.3.20 for Windows. Thematic analysis was applied to look for recognizable topics and patterns in the interviews and focus group data. Thematic analysis is suitable to investigate a specific a priori objective (e.g., understanding barriers and facilitators of health behavior adoption) with a predefined sample.^32^ First, two researchers (EB, IS) independently coded the transcripts inductively (sentence level open coding), meaning that codes were solely created based on what was found in the data in order to ensure no themes were lost with deductive analysis. After coding a transcript, the researchers discussed the open codes to reach a consensus. Next, to create broader categories, codes were organized and grouped in a process called axial coding.^33^ Subsequently, to conceptualize these categories in terms of types of health behavior influences, the researchers used a deductive approach by mapping these categories into the predefined domains and constructs of the TDF. Any discrepancies in the analysis between EB and IS were discussed until reaching a consensus. Consensus could be reached by keeping the original research question in mind and by thoroughly studying the context of the quotation. A third independent person could be consulted when these discrepancies remained. An illustrative example of the coding process is displayed in Figure 2.

## 3 RESULTS

### 3.1 Sample characteristics

In total, 22 participants were approached and agreed to participate in the individual interviews, while only 20 were actually interviewed (two no‐shows). After extensive discussion by two researchers (EB, IS), it was concluded that code saturation had been reached after one focus group and 20 interviews in total as no new codes emerged from subsequent interviews. Therefore, no further recruitment was conducted. Table 2 shows the sample characteristics of the childhood cancer survivors participating in the focus group and those interviewed individually. Overall, males and females were equally represented. All interview participants were Dutch, whereas almost half of the focus group participants originated from Eastern Europe. The median age of the participants was 30 years, and the majority of all participants had no partner or children living at home. Most of the participants had a paid job and only two focus group participants were considered as having a lower education level, that is, no schooling completed up to and including lower vocational education. Leukemia was the most frequent childhood cancer diagnosis, followed by malignant bone tumors. Median time since diagnosis was shorter in the focus group compared to the interview group (14 vs. 20 years, respectively) and only half of the focus group was currently receiving survivorship care. Regarding health behaviors, most childhood cancer survivors participating in the interviews considered their diet to be healthy and believed they participated in enough physical activity. In addition, a few of the childhood cancer survivors mentioned being smokers and/or (soft) drug users. Lastly, only a small number of interview participants mentioned consuming more alcohol per week than recommended by the Dutch Health Council, which was one drink a day for both women and men (Table 2).^35^

**TABLE 2:** Sample characteristics of childhood cancer survivors participating in telephone interviews or a focus group.^a^

	Focus group participants (*n* = 12)	Interview participants (*n* = 20)
Female gender	7	8
Country of birth		
Western Europe (the Netherlands)	1	20
Western Europe (other)	3	‐
Eastern Europe	5	‐
Northern Europe	2	‐
Southern Europe	1	‐
Age (years)^b^	29 (21–39)	30.5 (22–46)
Having a partner (yes)	2	7
Having children living at home (yes)	1	3
Having a paid job (yes)	7	17
Educational degree^c^		
High	7	10
Middle	3	10
Low	2	‐
Category of childhood cancer diagnosis^34^		
Leukemia	5	7
Central nervous system tumors and miscellaneous intracranial and intraspinal neoplasms	1	3
Brain tumors	‐	3
Other intracranial and intraspinal neoplasms	1	‐
Lymphomas and reticuloendothelial neoplasms	‐	2
Germ cell, trophoblastic, and other gonadal neoplasms	2	‐
Soft tissue sarcomas	‐	2
Malignant bone tumors	3	3
Renal tumors	1	2
Other and unspecified	‐	1
Years since completion treatment^b^	14 (3–28)	20 (9–40)
Receiving survivorship care	6	20
Perceiving having a healthy diet (yes)^d^	‐	6
Perceiving having enough physical activity (yes)^d^	‐	7
Non‐smoker (yes)^d^	‐	7
Non‐drug user (yes)^d^	‐	7
No excessive alcohol user (yes)^d^	‐	8

^a^ Data are shown as number unless otherwise indicated.

^b^ Data are shown as median (range).

^c^ Low education: no education, only primary school, secondary special education, practical education, or lower vocational education completed; middle education: preparatory secondary vocational education/general secondary education, secondary vocational education, higher general secondary education, or pre‐university education completed; high: higher professional education, or university completed.

^d^ For privacy reasons not asked at the focus group.

### 3.2 TDF domains

**FIGURE 3:**
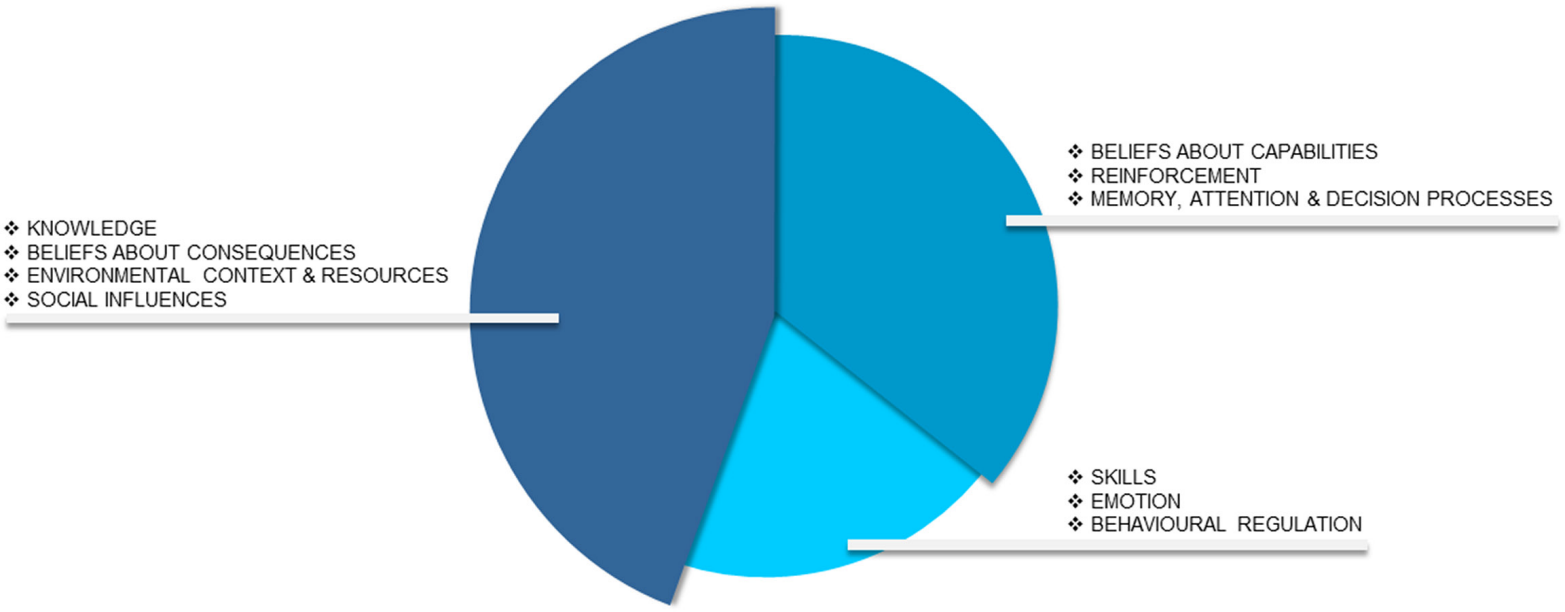
Domains of the Theoretical Domains Framework regarding barriers and facilitators of adopting and maintaining healthy behaviors in childhood cancer survivors participating in a focus group or individual interview, ordered by dominance.

In total, 10 out of 14 TDF domains were found to be relevant to the primary aim of this study. These domains will be described in more detail in the following sections. Barriers and facilitators regarding the TDF domains “Knowledge,” “Beliefs about consequences,” “Environmental context and resources,” and “Social Influences” were mentioned in all interviews and focus group (Figure 3). For this reason, these domains were considered as dominant. Barriers and facilitators related to the domains “Beliefs about capabilities,” “Reinforcement,” and “Memory, attention and decision processes” were mentioned in at least 19 out of the 21 conversations and were regarded as the second most dominant domains. Lastly, least dominant domains were “Skills,” “Emotion,” and “Behavioral regulation” with barriers and facilitators mentioned in 9–12 conversations. All relevant domains and the most significant barriers and facilitators corresponding to these domains are summarized in Table 3. Illustrative quotes of these findings are displayed in the text.

**TABLE 3:** Summary of key barriers and facilitators of adopting healthy behaviors in childhood cancer survivors.

Domain	Barrier	Facilitator
Knowledge^a^	Healthcare professionals providing insufficient knowledge on importance of health behaviors in childhood cancer survivors	Knowledge of importance health behaviors for childhood cancer survivor population
		Healthcare professionals providing knowledge on importance of health behaviors for childhood cancer survivors
		Healthcare professionals providing knowledge on how to engage in healthy behaviors
		Knowledge of family/friends/yourself on healthy behaviors
Skills		Learning how to deal with physical limitations when wanting to engage in physical activity
Beliefs about capabilities	Competence negatively influenced by physical limitations	Self‐discipline increasing perceptions of competence in health behavior adoption
		Self‐confidence increasing perceptions of competence in health behavior adoption
		Willpower increasing perceptions of competence in health behavior adoption
		Professional support increasing perceptions of competence in health behavior adoption
		Belief that healthy behaviors should be bearable to maintain
Beliefs about consequences^a^		Physical health benefits as consequences of healthy behaviors
		Long‐term health benefits as consequences of healthy behaviors
Reinforcement	Lower motivation to engage in healthy behaviors by personal related aspects	Positive reinforcement by personal‐related incentives
		Positive reinforcement by social/societal incentives
		Positive reinforcement by sport activity‐related incentives
		Positive reinforcement by distal rewarding of health behaviors
		Positive reinforcement by proximal rewarding of health behaviors
Memory, attention, and decision processes		Healthy behaviors due to conscious decision‐making
		Healthy behaviors embedded in memory
Environmental context and resources^a^	Lack of available time for healthy behaviors	Available professional support to stimulate healthy behaviors
		Work environment stimulating healthy behaviors
		Social environment positively influencing healthy behaviors
Social influences^a^	Unhealthy behaviors by people in close environment	Healthy behaviors of people in close environment
	Lack of social support in adopting healthy behaviors	Social support by people in close environment stimulating healthy behaviors
	(Social) Media stimulating unhealthy behaviors	(Social) Media stimulating healthy behaviors
		Dealing with negative influences from people in social environment
Emotion	Stress negatively affecting healthy behaviors	
Behavioral regulation		Good planning to maintain healthy behaviors

^a^ Dominant domain of the Theoretical Domains Framework based on high frequency of occurrence in interviews or focus group.

### 3.3 Knowledge

In terms of the domain “Knowledge,” many childhood cancer survivors expressed a desire to acquire information on why healthy behaviors are especially important for them. Healthcare professionals providing them with this knowledge was perceived as helpful, but some childhood cancer survivors indicated that this information was not always provided by healthcare professionals.

But nobody is explaining to you why it's good to exercise and why you should do it and nobody explained why you should have a good diet or why you shouldn't smoke or why you shouldn't drink too much alcohol, they [healthcare professionals] are just checking if you're doing it or not. (survivor #7, female, 24y)

The need for healthcare professionals to provide this knowledge on modifiable late effects risks and health behaviors was mentioned several times as a potential facilitator for adopting or maintaining healthy behaviors.

It would be nice if you were offered some help, that you would be offered information that is known after certain treatments, certain chemo or radiation, which can be helpful. That you can think about what shortages you can run into, what kind of movement can be helpful. (survivor #24, female, 26y)

Childhood cancer survivors also reported that healthcare professionals should use an appropriate approach with information being provided in the right way with concrete information and a tailored approach to the individual. On the other hand, several childhood cancer survivors preferred obtaining knowledge on healthy behaviors by themselves without a healthcare professional interfering. In general, a good common sense on health behaviors was perceived by childhood cancer survivors as a facilitator to adopting or maintaining healthy behaviors.

I think it really helps to be knowledgeable about what healthy eating means. (survivor #25, male, 32y)

### 3.4 Beliefs about consequences

Concerning the domain of “Beliefs about consequences,” childhood cancer survivors regarded health benefits as positive prospects of healthy behaviors.

I also think it's important to live a healthy life as my body already has some sort of disadvantage. I had cancer at a very early age, so I want to be able to function as optimally as possible for as long as possible. (survivor #24, female, 26y)

These prospects, including physical health and long‐term health, were therefore considered facilitators of pursuing healthy behaviors. Physical health benefits were mostly related to feeling more energized and fit, whereas long‐term health benefits were related to a good quality of life with few physical complaints.

I know what will happen if I don't [complying with healthy behaviors]. That I might become weaker or that I might have less stamina, or that I might—well, yes—have health problems because I eat less varied. I see the consequences of that. I choose not to have those consequences as I want to become eighty. So, therefore, I really have to maintain this lifestyle. (survivor #30, male, 27y)

### 3.5 Environmental context and resources

For most childhood cancer survivors, a profound resources‐related barrier to healthy behaviors was the lack of available time to execute healthy behaviors such as cooking healthy meals or performing physical activities.

I work 40 hours a week so, in that respect, when I come home, I'm just tired from work. So often I don't really feel like exercising, so to speak. (survivor #15, male, 30y)

On the other hand, professional support was mentioned to be a resource‐related facilitator which can enable them to adopt healthier behaviors. This could include dieticians or physiotherapists, but also psychologists or other coaches who can provide psychosocial support. This was especially perceived as helpful for childhood cancer survivors in gaining confidence, dealing with their cancer history, and sticking to adopting healthier behaviors.

A conversation [with a healthcare professional] in which you have to give some accountability for the things you do and why you did them. That actually always makes me feel like I have to do this [complying with healthy behaviors] otherwise I will have to tell you that it didn't work out. (survivor 26#, male, 31y)

The participants also voiced that a working place can also be a good contributor in supporting healthy behaviors when promoting a healthier diet and/or higher levels of physical activity by distinct means or activities.

If you sit behind your desk all day, of course that won't help you. So, we were made very conscious [on this] last week at work […] It can also help if a company is working on it itself. (survivor #13, male, 31y)

In addition, the direct (social) environment of childhood cancer survivors was found to be influencing behaviors as well. For instance, some childhood cancer survivors indicated that living on their own helped with making the right healthy choices.

### 3.6 Social influences

When considering the domain of “social influences,” several childhood cancer survivors indicated perceiving it as demotivating to pursue healthy behaviors when families and/or friends failed to comply with healthy behaviors themselves.

I also have friends and family who are also active with sports and health, so that … That makes it then quite easy, to go along with that, or to motivate others from within myself. That is also a positive thing that helps me to go. (survivor #31, male, 38y)

Many childhood cancer survivors preferred being in an environment where others followed healthy behaviors as well. However, other childhood cancer survivors learned how to deal with negative influences from people in their social environment, making that a facilitating factor for adopting or maintaining healthy behaviors.

In addition, (social) media was mentioned to be both a barrier and facilitator as both unhealthy and healthy products and recipes are often promoted in the media. One of the disadvantages of (social) media was believed to be the tempting images or videos of unhealthy food depicted.

Subconsciously, I think that it also…influences [healthy behaviors] very much. On the one hand when they advertise all kinds of tasty, unhealthy things. Then you think “oh I would like to try that.” (survivor #20, female, 37y)

In contrast, an increase in awareness of the importance of healthy behaviors was said to be a facilitator. A major complicating barrier for adopting healthier behaviors was said to be the lack of social support of people in the close environment.

I mean, I am also supported by my family in the fact that I want to lose weight for example, and they respect that and they try to motivate me in that as well. My boyfriend tries to motivate me in that too, but he is not going to participate in what I do. (survivor #19, female, 23y)

Likewise, having a solid support system, helped childhood cancer survivors in reaching their health goals.

### 3.7 Skills

Some childhood cancer survivors indicated that they had physical limitations due to late effects, which hindered them from performing certain physical activities.

Well, tired easily, sore muscles that didn't recover quickly. […] And that actually hinders you from exercising […]. I also quit for a while and because of work I also quit exercising now. (survivor #29, male, 44y)

On the other hand, learning how to deal with those physical limitations and to find a suitable and enjoyable alternative for physical activity, was found to be an enabling factor to behave healthy.

### 3.8 Beliefs about capabilities

According to several childhood cancer survivors, their competence to perform physical activities was also negatively affected by physical limitations caused by late effects of cancer:

I suffer from late effects in daily life. I experience quite a lot of fatigue and pain and because of that I notice that I am very limited in energy, so exercise and sports are quite difficult for me. (survivor #24, female, 26y)

Notwithstanding, childhood cancer survivors often argued that self‐discipline, self‐confidence, and willpower were important helping factors when it comes to succeeding in reaching and sustaining healthier behaviors.

I can't say that it always works out well, but I can have good discipline. (survivor #24, female, 26y)

Some childhood cancer survivors perceived that their self‐efficacy was insufficient and hence they would like professional support to increase their competence in adopting healthy behaviors. To be able to maintain healthy behaviors in a more long‐term perspective, some childhood cancer survivors suggested that healthy behaviors should be flexible. For instance, this can be achieved by allowing yourself an unhealthy treat from time to time.

Food is one of the pleasures in life. And yes, moderate alcohol consumption too. So yes, I try to find some balance in that. (survivor #22, male, 46y)

### 3.9 Reinforcement

Regarding the TDF domain “Reinforcement,” childhood cancer survivors perceived a low intrinsic motivation for health behaviors or a preference for unhealthy food options as barriers to healthy behaviors.

I know perfectly well when I'm in the grocery store that I shouldn't buy this bag of cocktail nuts. But, yeah, […], then you think: “But yeah, it is tasty though.” (survivor #22, male, 46y)

As mentioned by several childhood cancer survivors, positive reinforcement of healthy behaviors could be reached by either personal‐, social‐, or sport activity‐related incentives. Personal incentives were mostly determined by intrinsic motivation for healthy behaviors.

To be an example for your children in healthy living, healthy eating. (survivor #20, female, 37y)

Social‐related incentives related to working with others toward a goal were important enablers of healthy behaviors as well. Concerning sport activity‐related incentives, performing sport activities according to their own preferences was often perceived as a motivator for physical activity. Reinforcement could also be established by rewards, both distally and proximally. Distal rewards were found to be related to health in the long‐term, whereas examples of proximal rewards were having a higher energy level and better‐fitting clothes. Allowing yourself a little treat after sports was also cited to be an enabling factor to comply with healthy behaviors.

Sports are great to do, but you don't always feel like it. Sometimes it is also cold and rainy, but after exercise, you feel satisfied, cause you have done it anyway. But also having a drink afterward on a terrace, that is a kind of reward that you create yourself. (survivor #25, male, 32y)

### 3.10 Memory, attention, and decision processes

Regarding the domain of “Memory, attention, and decision processes,” it was believed that decision‐making on health behaviors was related to their own consciousness by many childhood cancer survivors.

In my opinion, it is also just a choice, so just being aware of what healthy eating is. […] That you just consciously buy fruit and vegetables in the supermarket. (survivor #15, male, 30y)

Acting on what is known about health behaviors makes it easier for childhood cancer survivors to comply with them. Healthy behaviors which were embedded in memory was reported as a facilitator as well.

Once it's part of me in a certain way, I never forget it. Then that's no problem. (survivor #32, male, 29y)

### 3.11 Emotion

Several childhood cancer survivors expressed that stress can be a hindering factor for them when adopting or maintaining healthy behaviors. For some childhood cancer survivors, stress resulted in losing excitement for physical activities, whereas, for other childhood cancer survivors, stress resulted in pleasure‐seeking of unhealthy behaviors.

If I really don't feel well, then diet is the very first thing I notice it with. Then I will eat a lot more and then I will actually act nonchalant about it. Then it doesn't really matter to me anymore that I've put on weight. Although I do regret it afterward. (survivor #19, female, 23y)

### 3.12 Behavioral regulation

Lastly, health behaviors could be regulated and maintained by making sure those behaviors align with the personal situation of a childhood cancer survivor. Therefore, proper planning of healthy behaviors was cited as a facilitator for health behavior adoption and maintenance.

So, what is very important for me is that I make some more time for myself in the weekends, so that I don't let all my time be consumed by social activities, because basically if I just have time for myself on weekends, then I live healthier too. (survivor #15, male, 30y)

## 4 DISCUSSION

This study was set out to explore barriers and facilitators to adopting healthy behaviors as perceived by childhood cancer survivors. A wide range of barriers and facilitators were identified which were most often related to the TDF domains “Knowledge,” “Beliefs about consequences,” “Environmental context and resources,” and “Social influences.”

In our study, knowledge emerges as an influencing factor for childhood cancer survivors in pursuing healthy behaviors. Understanding why healthy behaviors are especially relevant for the childhood cancer survivor population is therefore crucial for health behavior adoption or maintenance. Healthcare professionals have a key role in providing this knowledge. The need for healthcare professionals to educate childhood cancer survivors on health behaviors was reflected in both our study as well as in a study by Pugh et al. with teenage and young adult cancer survivors preferring to be given education on the benefits of a healthy lifestyle.^35^ Well‐educated healthcare professionals with both knowledge of late effects of cancer and health behaviors are therefore a requirement to ensure proper health behavior education of childhood cancer survivors in the survivorship care setting. Childhood cancer survivors also expressed relying on health behavior knowledge from family and friends. This pattern was also observed in a study by Meer et al., in which family and friends were among the most frequently reported sources of health behavior information.^36^ However, when relying on family and friends for health behavior advice, people should be cautious with potentially incorrect information.

We found that the availability of professional support was a relevant facilitator for health behavior adoption in childhood cancer survivors, which was not limited to either dietary or physical activity support, but also to psychosocial support. Childhood cancer survivors may be dealing with processing their cancer history and/or may experience multiple psychosocial burdens due to late effects of cancer which can impede successful health behavior change. Therefore, psychosocial support for this population is recommended and should be included when developing new lifestyle interventions for this population.^37^ This will enable childhood cancer survivors to focus on their health behaviors without the influences of negative emotions. Regarding environmental factors, this study showed that the workplace of childhood cancer survivors can have a positive influence on healthy behaviors. For this reason, the implementation of facilities at workspaces aiming at increasing healthy behaviors of employees may have the potential to improve healthy behaviors also among the general population. Consistent with other performed studies, a lack of time was perceived as a major barrier to adopting and maintaining healthy behaviors.^24^, ^25^, ^35^ Due to school activities, work, or care for children, people often fail to perform enough physical activity and/or to shop and prepare healthy meals. Though this is not an easy problem to solve, personalized professional support can help childhood cancer survivors in coping with time constraints and in planning healthy behaviors.

Consistent with a study by Arroyave et al., childhood cancer survivors in this study indicated unhealthy behaviors of others to be demotivating when trying to adopt healthier ones themselves.^24^ Conversely, learning how to cope with the negative influences of the social environment can help overcome this barrier. In the current study, the lack of support in the survivor's social environment was also reported as a barrier to health behavior adoption. As Pugh et al. mentioned in a study among teenage and young adult survivors, peer and social support can help to increase confidence and self‐efficacy, and hence, the chance of successful health behavior adoption.^35^ Lastly, although (social) media offers a range of opportunities to increase health consciousness and to promote healthy behaviors, this study illustrated that it is key for websites and social influencers to provide readers and followers with correct health‐stimulating messages. Only when this precondition is met, can (social) media have a meaningful health‐promoting potential.

A strength of this study is the data originating from the focus group consisting of participants originating from all regions in Europe, including countries with established survivorship care centers with follow‐up of adult childhood cancer survivors as well as countries lacking such follow‐up. However, as for most participants, the focus group was not conducted in their native language, some participants may have had some trouble with the language barrier. Furthermore, this study is limited by the lack of information on cancer treatment for the included childhood cancer survivors. Other barriers could be expected with childhood cancer survivors who are dealing with a lot of late effects of their specific cancer treatment (e.g., craniospinal radiotherapy leading to disturbed hormone axes). Another limitation of this study is the lack of diversity in the socio‐economic status of the participants. In this study, the majority of the participants had a relatively high socio‐economic status. As research has shown that the socio‐economic status and health behaviors are strongly correlated, it would have been interesting to explore the barriers and facilitators of childhood cancer survivors with a low socio‐economic status and potential worse health behaviors.^38^ In addition, our sample may not be representative for the whole childhood cancer survivor population concerning socio‐economic status. Lastly, a complicating factor of this study lies in the fact that it focused on multiple (health) behaviors. As the TDF is designed for singular behaviors, barriers, and facilitators would have been more specific when focusing on one behavior. Notwithstanding these limitations, this study does provide an informative and clinically relevant overview of barriers and facilitators focusing on a more complete assessment of childhood cancer survivors' health behaviors.

Taken together, this study has identified a need for support and education in health behaviors and its relevance for the survivor population provided by healthcare professionals in order to facilitate health behavior adoption in childhood cancer survivors. In addition, the lack of time to perform healthy behaviors was a profound barrier to pursue healthy behaviors in childhood cancer survivors. A key policy priority for health policymakers and healthcare professionals involved in survivorship care should therefore be to incorporate health behavior support in standard follow‐up care for childhood cancer survivors as well as to develop interventions, personalized interventions accounting for the personal‐, environmental‐ and social‐related barriers childhood cancer survivors experience when adopting or maintaining healthy behaviors.

## AUTHOR CONTRIBUTIONS

**Eline Bouwman:** Conceptualization (equal); data curation (lead); formal analysis (lead); investigation (lead); methodology (equal); project administration (equal); visualization (equal); writing – original draft (lead). **Saskia M. F. Pluijm:** Conceptualization (equal); funding acquisition (equal); investigation (supporting); project administration (equal); supervision (equal); writing – review and editing (lead). **Iridi Stollman:** Formal analysis (lead); investigation (equal); validation (equal); visualization (lead); writing – review and editing (equal). **Vera Araujo‐Soares:** Conceptualization (lead); funding acquisition (supporting); methodology (lead); writing – review and editing (lead). **Nicole M. A. Blijlevens:** Supervision (supporting); writing – review and editing (supporting). **Cecilia Follin:** Writing – review and editing (supporting). **Jeanette F. Winther:** Funding acquisition (equal); writing – review and editing (equal). **Lars Hjorth:** Funding acquisition (equal); writing – review and editing (equal). **Tomas Kepak:** Funding acquisition (equal); writing – review and editing (supporting). **Katerina Kepakova:** Writing – review and editing (supporting). **Leontien C. M. Kremer:** Funding acquisition (lead); writing – review and editing (supporting). **Monica Muraca:** Funding acquisition (equal); writing – review and editing (equal). **Helena J. H. van der Pal:** Funding acquisition (equal); project administration (equal); resources (equal); writing – review and editing (supporting). **Carina Schneider:** Writing – review and editing (supporting). **Anne Uyttebroeck:** Funding acquisition (equal); project administration (equal); writing – review and editing (supporting). **Gertrui Vercruysse:** Writing – review and editing (supporting). **Rod Skinner:** Conceptualization (equal); funding acquisition (equal); writing – review and editing (equal). **Morven C. Brown:** Conceptualization (lead); funding acquisition (supporting); methodology (lead); writing – review and editing (lead). **Rosella P. M. G. Hermens:** Funding acquisition (equal); methodology (lead); project administration (equal); supervision (lead); validation (lead); writing – review and editing (lead). **Jacqueline J. Loonen:** Conceptualization (equal); funding acquisition (equal); project administration (equal); resources (lead); supervision (lead); validation (lead); writing – review and editing (lead).

## FUNDING INFORMATION

This work was supported by the European Union's Horizon 2020 Framework Program (Grant Number 824982). The funder had no role in study design, data collection, data analysis, data interpretation, or in writing the report. The material presented and views expressed here are the responsibility of the author(s) only. The EU Commission takes no responsibility for any use made of the information set out.

## CONFLICT OF INTEREST STATEMENT

The authors declare that they have no known competing financial interests or personal relationships that could have appeared to influence the work reported in this paper.

## ETHICS STATEMENT

The procedures of this study were approved by METC Oost‐Nederland (case number 2019‐5630) and conforms to the principles of the Declaration of Helsinki (Fortaleza, Brazil, Version 2013) and the Dutch Medical Research Involving Human Subjects Act.

## PATIENT CONSENT STATEMENT

Written informed consent was obtained from all participants.

## Supporting information


Table S1.

## Data Availability

The PanCareFollowUp project aims to comply with all the four FAIR principles and to share individual de‐identified data upon request. At moment of writing, both the PanCareFollowUp project as well at the Radboud University Medical Center are working on establishing the conditions and means to share the data. Requests for de‐identified data should be made to the corresponding author (EB).
