# Supplementary material for: Perceived barriers and facilitators to health behaviors in European childhood cancer survivors: A qualitative PanCareFollowUp study
Source: Cancer Med. 2023 Apr 7;12(11):12749–64. doi: 10.1002/cam4.5911 (PMC10278475; doi:10.1002/cam4.5911)
Supplement: Supplementary file 1 — Table S1. [file CAM4-12-12749-s001.docx]

**Supporting Information**

**Table S1**. Topic guide focus group and interviews

| **Topic/domains** |  | **Questions** | **Probes** |
| --- | --- | --- | --- |
| *Lifestyle in general* |  | 1. *Can you tell me what you consider as a healthy lifestyle?* 2. *Can you tell me something about your lifestyle?^†^* |  |
| *General barriers and facilitators to healthy behavior adoption and/or maintenance* |  | 1. *Which bottlenecks or problems could you think of or have you experienced when keeping a/adopting to a healthy lifestyle?* 2. *Which factors could support you or did you experience in successfully adopting to a healthy lifestyle?* | - Physical barriers, personal barriers, etc. - Supporting family, knowledge, etc. |
| *Knowledge* |  | 1. *What do you know about why it is important to keep a/adopt to healthy behaviors?* 2. *To what extent do you think knowing why healthy behaviors are important contributes to adopting or maintaining healthy behaviors?* 3. *To what extent do you think knowledge about healthy behaviors contributes to adopting or maintaining healthy behaviors?* |  |
| *Social role and identity* |  | 1. *Who do you think is responsible for your lifestyle? (prompt - to what extent do you think your lifestyle behaviours are the responsibility of your current health care professional?)* | - Social identity |
| *Social influences* |  | 1. *Which attitudes do your family and/or friends hold about a healthy lifestyle? (prompt – are these attitudes conflicting with your own attitudes/beliefs about a healthy lifestyle?)* 2. *How do you think your family and/or friends influence you if they adopt their lifestyle?* 3. *How do you think you are influenced by media about a healthy lifestyle?* | - Social pressure - Social/group norms - Social comparisons   Social support |
| *Skills* |  | 1. *Can you tell me something about the physical and/or mental skills you need to adopt to/keep a healthy lifestyle?* | - Physical/mental skills - Competence - Ability |
| *Beliefs about capabilities and optimism* |  | 1. *Can you tell something about your confidence level to adopt to/ keep a healthy lifestyle?* 2. *What do you think about your (mental) ability to adopt to or to keep a healthy lifestyle on your own? (prompt - do you need help in this? Do you think you are able to express your needs in this case?)* | - Self-confidence - Perceived competence - Self-efficacy - Empowerment - Optimism - Pessimism |
| *Environmental context and resources* |  | 1. *Can you tell something about the necessary resources/materials you need to adopt to/ keep a healthy lifestyle?* | - Resources/ material resources |
| *Behavioral regulation* |  | 1. *What do you think is necessary for you to keep a healthy lifestyle once succeeded?* | - Action planning - Long-term reaching goals |
| *Emotions* |  | 1. *How do you think that your feelings affect whether you want to adopt to a healthy lifestyle or not?* | - Stress - Anxiety - Fear - Depression - Positive/ negative affect   Burn-out |
| *Reinforcement* |  | 1. *What can motivate you to adopt to a healthy lifestyle?* | - Rewards (proximal/distal, valued/not valued)   Incentives |
| *Beliefs about consequences* |  | 1. *How do you think a healthy lifestyle will affect your daily life and health?* | - Beliefs   Outcome expectancies |
| *Memory, attention, and decisions process* |  | 1. *How easy or difficult is it to remember how you can adopt to or keep a healthy lifestyle?* 2. *Can you tell something about your decision-making processes when it comes to chose for healthy/unhealthy options?* | - Memory - Decision making |
| **Closing**   \| Is there anything else you would like to add about providing lifestyle advice to survivors? \| \| --- \| | | | |

^†^ For privacy reasons not asked at the focus group.
